# Clinical and Dermoscopic Patterns of Basal Cell Carcinoma and Its Mimickers in Skin of Color: A Practical Summary

**DOI:** 10.3390/medicina60091386

**Published:** 2024-08-24

**Authors:** Emmanouil Karampinis, Konstantina-Eirini Georgopoulou, Elli Kampra, Efterpi Zafiriou, Aimilios Lallas, Elizabeth Lazaridou, Zoe Apalla, Biswanath Behera, Enzo Errichetti

**Affiliations:** 1 Second Dermatology Department, School of Health Sciences, Aristotle University of Thessaloniki, 54124 Thessaloniki, Greece; koneirgeo@gmail.com (K.-E.G.); bethlaz@auth.gr (E.L.); zoimd@yahoo.gr (Z.A.); 2 Department of Dermatology, Faculty of Medicine, School of Health Sciences, University General Hospital of Larissa, University of Thessaly, 41110 Larissa, Greecezafevi@o365.uth.gr (E.Z.); 3 First Dermatology Department, School of Health Sciences, Aristotle University of Thessaloniki, 54124 Thessaloniki, Greece; emlallas@gmail.com; 4 Department of Dermatology and Venereology, All India Institute of Medical Sciences (AIIMS), Bhubaneswar 751019, Odisha, India; biswanathbehera61@gmail.com; 5 Institute of Dermatology, Department of Medical Area, University of Udine, 33100 Udine, Italy; enzoerri@yahoo.it

**Keywords:** dermoscopy, skin of color, basal cell carcinoma, skin cancer, cutaneous neoplasms, skin infections, cutaneous dermatoses

## Abstract

The diagnosis of basal cell carcinoma (BCC) in dark phototypes can be a challenging task due to the lack of relevant clues and its variable presentation. In this regard, there is growing evidence that dermoscopy may benefit the recognition of BCC even for skin of color (SoC). The objective of this review is to provide an up-to-date overview on clinical and dermoscopic patterns of BCC in SoC, also comparing such findings with those of the main clinical mimickers reported in the literature. A comprehensive search of the literature through the PubMed electronic database was carried out in order to identify papers describing the clinical and dermoscopic features of BCC in dark phototypes (IV–VI). By finding macroscopic clinical presentations of BCCs in SoC patients and any possible clinical mimickers considered in the retrieved papers, we built a differential diagnosis list and analyzed the dermoscopic findings of such conditions to facilitate the diagnosis of BCC. BCC in darker skin may present as pigmented nodular lesions, pigmented patches or plaques, ulcers, erythematous nodular lesions, erythematous plaques or patches, or scar-like lesions, depending on its subtype and body site. The differential diagnosis for BCC in patients with SoC includes squamous cell carcinoma, melanoma, nevi, adnexal tumors and sebaceous keratosis. Additionally, it differs from that of Caucasians, as it also includes lesions less common in fair skin, such as dermatosis papulosa nigra, melanotrichoblastoma, and pigmented dermatofibrosarcoma protuberans, and excludes conditions like actinic keratosis and keratoacanthoma, which rarely appear in darker skin. The resulting differences also include infectious diseases such as deep cutaneous mycosis and inflammatory dermatoses. The most prevalent differentiating dermoscopic feature for BCC includes blue, black and gray dots, though arborizing vessels still remain the predominant BCC feature, even in dark phototypes. Diagnostic approach to BCC in dark-skinned patients varies due to the prevalence of dermoscopy findings associated with hyperpigmented structures. Clinicians should be aware of such points of differentiation for a proper management of this tumor in SoC.

## 1. Introduction

Basal cell carcinoma (BCC) is one of the most common malignant skin tumors in dark phototypes [1]. However, its recognition may not be so straightforward in this group of patients as relevant clinical clues may be lacking/less evident, such as translucency and telangiectasias. Additionally, BCCs may display a wider range of presentations from a morphological point of view compared to fair-skinned patients, also being mistaken for a non-tumoral dermatosis [1]. Over the last few years, several studies have shown the possible usefulness of dermoscopic examination in supporting the diagnosis of skin tumors in dark phototypes, especially BCC [2]. Reviews on skin of color (SoC), focusing on dermoscopy of cutaneous neoplasms [3], hair diseases [4], skin infections [5], and inflammatory dermatoses [6], have revealed differences in the frequency of certain dermoscopic traits between Caucasian and SoC patients. In the emerging field of artificial intelligence, AI programs also provide different differential diagnoses for the same skin condition when presented on brown or dark skin [7]. In this regard, dermoscopy should be considered as an additional piece of the diagnostic puzzle to couple with anamnestic and clinical data in order to significantly increase accuracy in the case of SoC patients.

Of note, the spectrum of “skin of color” (SoC) encompasses several races, e.g., people of African, Asiatic, Hispanic, and Caribbean descent, with possible variability. SoC is often mistakenly viewed as a homogeneous group, whereas it actually consists of significantly diverse populations in terms of race-specific characteristics. It is also important to note that racial groups, particularly Hispanic and Asian, include individuals with fair as well as dark skin types (traditionally phototypes IV–VI), making the ethnic skin categorization even more complicated [8].

The aim of the present review paper was to provide a comprehensive overview of the clinical and dermoscopic presentation of BCC in SoC, also comparing such findings with those of the main clinical mimickers and reporting possible variations according to patient’s race.

## 2. Materials and Methods

A comprehensive search of the literature was performed through the PubMed electronic database from inception to 1 November 2023 using the following search terms: “basal cell carcinoma” OR “basalioma” OR “basal cell epithelioma” AND “skin of colour” OR “African” OR “Indian” OR “Asian”. Titles, abstracts, and full texts were screened by two independent reviewers to select articles reporting either clinical or dermoscopic features of BCC in dark skin (Fitzpatrick’s phototypes IV–VI); instances without histological diagnosis, non-English articles, reviews, personal opinions/editorials and duplicates were ruled out. A manual search was also carried out by analyzing the reference sections of all relevant studies or reviews on the topic. If information on the skin phototype was not provided, a decision on inclusion was made based on whether the title/abstract/full text reported that the manuscript concerned “dark skin” or “skin of color” and for single instances also based on the attached figures. The race of the patient was also recorded when specified.

As a second step, we performed a further PubMed search to identify BCC-like clinical and dermoscopic clues (if any) of the conditions were considered in the differential diagnosis in retrieved articles on BCC in SoC. Search terms included the name of the condition AND “skin of colour” OR “African” OR “Indian” OR “Asian”. For both BCC and mimickers, clinical features, histological subtype (for BCC), dermoscopic findings, histological background (if reported), dermoscopic setting (polarized vs non-polarized and magnification degree), skin type of the patient (if available), race (if reported), and number of cases were assessed and summarized. When it comes to dermoscopic data, we ruled out single case-reports to ensure a higher level of evidence. All of the included studies were classified according to standard definitions for diagnostic accuracy studies and their level of evidence was assigned based on The Oxford 2011 Levels of Evidence.

## 3. Results

Considering the macroscopic clinical presentation of BCCs, we found 42 studies addressing BCC in skin of color. Most of them were clinical case reports (28 studies [9,10,11,12,13,14,15,16,17,18,19,20,21,22,23,24,25,26,27,28,29,30,31,32,33,34,35,36]), with also 13 retrospective case series and [37,38,39,40,41,42,43,44,45,46,47,48,49], 1 cross-sectional analysis [50] and 1 prospective comparative analysis [51].

The main morphology reported in the literature was hyperpigmented nodular BCC (50% of the studies identified referenced this subtype of BCC, with it being the prevailing form in case series studies as in Supekar et al. [42]), followed by ulcerated lesions (33%) and plaques (17%). Also, in many cases, the nodular and ulcerative subtype co-existed as in the case report of Abudu B. [35] and in the cross-sectional analysis of Kumar S. [50]. The neck and head were the most common localizations [38] and female patients turned out to be more involved according to the largest study [51]. In terms of histological subtypes, nodular and superficial BCC were the most common, with adenoid [14,15] and morpheaform [16] variants detected in a minority of cases. However, in one retrospective study the predominant histological subtype included micronodular and microcystic [37]. Concerning ethnicity, the most represented group in the case series were Asian populations (Koreans, Japanese, Chinese) (50%, 6/12) followed by Indian (41.7%, 5/12) and African (8.3%, 1/12), while the case reports featured Indian individuals at 17.9% (5 out of 28), African American individuals at 14.3% (4 out of 28), and Hispanic individuals at 17.9% (5 out of 28) [42,43,44,45,46], with the rest of the case reports reporting skin of color patients without ethnicity specification.

Differential diagnosis (BCC mimickers) is crucial for diagnosis. In Table 1, we present studies which rely on pre-existing differential diagnoses of the studies’ authors, minimizing the potential for bias that could be introduced by our own interpretations or preferences. This approach leads to a more objective and balanced analysis.

Among the retrieved articles on BCC, there were 22 studies encompassing a range of potential differential diagnoses on clinical grounds (Table 1), including neoplasms, skin infections and inflammatory dermatoses. The most frequent mimicker is squamous cell carcinoma (SCC) (59.1%, 13/22) followed by melanoma (36.4%, 8/22) and seborrheic keratosis (27.3%, 6/22). Studies focusing on eyelid BCCs in SoC patients usually included a differential diagnosis of sebaceous gland carcinoma and adnexal neoplasms such as apocrine hidrocystomas along with SCC. Benign neoplasms (e.g., nevi, hemangioma and benign appendage lesions), malignancies (e.g., melanoma and pigmented dermatofibrosarcoma protuberans) and deep fungal infections (especially chromoblastomycosis) were all included in the main differential diagnoses when it comes to nodular BCC. In cases of infiltrating BCC in individuals with darker skin tones, SCC was consistently included in the differential diagnosis, while adenoid BCC was associated with various potential diagnoses such as lichen atrophicus and extramammary Paget disease. Additionally, only one instance of morpheoform BCC was identified in individuals with darker skin tones, with suggested alternative diagnoses including melanoma, seborrheic keratoses, or nevus sebaceous. The suggested race-specific differences in the differential diagnosis were not present in dark-skinned individuals.

Moving to the dermoscopic features of BCC, Manci et al. frequently noted the absence of a typical background pigmentation or network, a milky-red region and an intensified normal background pigmentation or network encircling the lesion [52]. Additionally, besides the lack of pigment network, the most prevalent findings found in another study included gray, black and blue dots [3]. Other minor/less common features of BCC reported in the literature are summarized in Table 2 and presented according to the clinical subtypes [2,3,52,53].

Clinically, in most studies, the nodular BCCs presented as a pigmented nodules and superficial BCC presented as a hyperpigmented plaque (Table 2) in medical bibliography. However, many skin lesion subtypes can present with the same clinical image. Table 3 illustrates that squamous cell carcinoma (SCC) and melanoma can mimic basal cell carcinoma (BCC) in individuals with darker skin tones across various subtypes. For instance, SCC may manifest as an ulcerated lesion or pigmented or red plaque, whereas melanoma can present as a nodular pigmented lesion, ulcerated lesion, or pigmented or red plaque. Dermoscopy may offer assistance in distinguishing between these conditions in such comparisons. Table 4 features the main dermoscopic clues of all considered mimickers according to a literature search on SoC while Table 5 includes a correlation between dermoscopy features and possible diagnosis.

**Figure 1:**
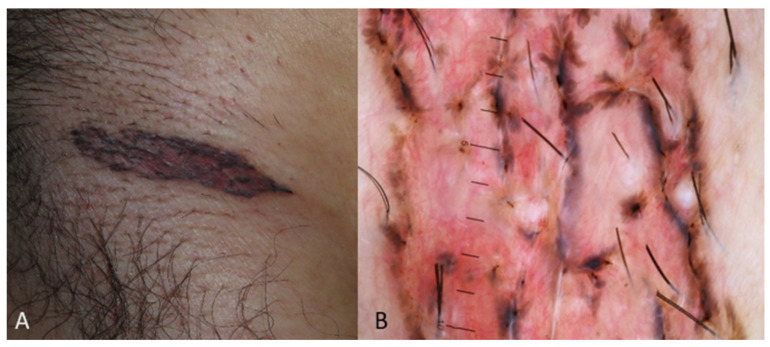
Presenting macroscopic and dermoscopic presentation of BCC in Indian patient. Image (**A**) showing a pigmented patch and image (**B**) indicating the respective dermoscopy image with the presence of structureless black or leaf-like areas in a pink stroma.

**Figure 2:**
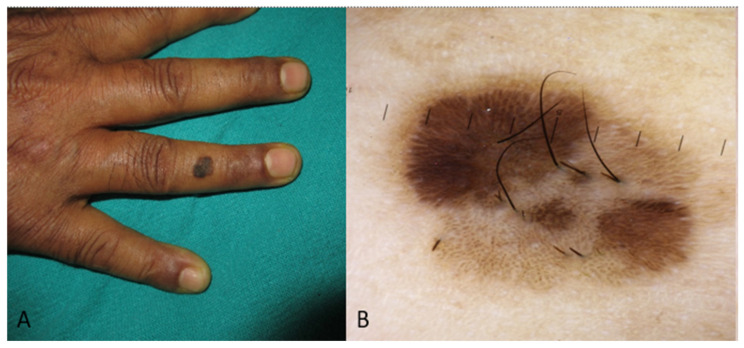
Presenting a sebaceous keratosis on the skin of the ring finger of a dark-skinned individual as a pigmented patch (similar presentation as BCC or acral melanoma) (Image (**A**)). Image (**B**) shows the differently tanned finger-like structures, which is dermoscopy characteristic of sebaceous keratosis.

**Figure 3:**
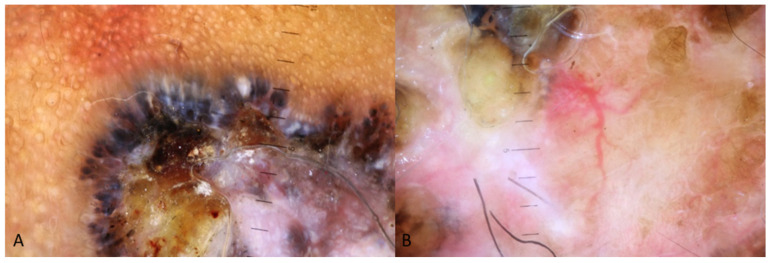
Presenting dermoscopy images of a nodular lesion-BCC in an Indian patient. Image (**A**) shows the pigmented structures on the periphery of the lesions with blue-white veil a centrally while in image (**B**) the arborising vessels are present on a pink-brownish stroma with co-existing white chrysalis structures.

Figure 1, Figure 2 and Figure 3 display some examples of BCC and clinical mimickers with dermoscopic differentiating clues.

## 4. Discussion

Diagnosing basal cell carcinomas (BCCs) in individuals with darker skin tones can present challenges, primarily stemming from variations in presentation and an extensive list of potential differential diagnoses. Additionally, the discrepancies in dermoscopic characteristics contribute to the difficulty, given that the majority of training in healthcare professions has historically focused on patients with lighter skin tones [2]. Comparing BCC differential diagnosis between Caucasians and SoC patients, most diseases co-exist in both categories such as skin appendage tumors as squamous cell carcinoma, while nevi and melanoma remain the main BCC mimickers. However, the BCC subtype (nodular, etc.) and BCC site (eyelid, etc.) can further affect the differential diagnosis thinking. The dermoscopy images in the general population and SoC populations demonstrate comparable dermoscopy patterns in a significant proportion. Nonetheless, distinctions arise, particularly in the prevalence of dermoscopy findings associated with hyperpigmented structures (as seen in BCCs), owing to variations in darker color pigmentation patterns. Also, it is worth mentioning that from the list of skin lesions that resemble BCC, actinic keratosis and keratoacanthoma are missing. This observation primarily arises from the fact that these lesions result from cumulative sun exposure, and in the case of individuals with dark skin, who are less prone to damage from sun exposure, such lesions are infrequently encountered [111,112]. It is also worth mentioning that the regression trait which was seen in Caucasian melanomas was not observed in SoC melanoma reviews, indicating that some dermoscopy indicators of certain skin diagnoses may be absent or less frequent when appearing on dark skin. Therefore, searching for regression to include melanoma and exclude BCC in a pigmented nodular lesion may not be as useful in SoC patients as in Caucasians.

Also, epidemiological reports should be taken into consideration as the correlation between BCC occurrence, site of the skin cancer, age and ethnic group. For example, in a prospective study comparing Caucasians and Asians in Singapore, the authors found that contrary to the Chinese population, BCC exhibited a higher frequency among younger Caucasians, mainly occurring to the trunk and upper limb. Additionally, nodular pigmented BCC was more prevalent on the head and neck of elderly Chinese individuals [51]. Despite variations in skin types and skin cancer prevalence among different ethnic groups, the gold standard for diagnosing skin cancer continues to rely on elements such as medical history, the clinical and dermoscopic characteristics of the lesion, and histopathological examination.

The main dermoscopic findings of BCC in SoC found in both reviews about skin cancer in the SoC population were well-circumscribed, pigmented ovoid areas; when large, they were described as blue, black and gray, while when they were smaller, they were described as blue, black and gray dots [2,3]. One of the two reviews focused on the further deviation of skin of color terminology, reporting both race-specific dermoscopy characteristics of BCC [2]. According to this review, the dermoscopy image of the BCC in most cases resembles the respective image of a pigmented BCC reported in Caucasians (maple-like leaves, radial wheel structures, etc.); the blue veil presented in SoC patients (Hispanics and dark-skinned patients) mainly confuse the clinicians regarding the direction of melanoma. Arborising vessels are also the dominant vessel pattern of BCCs in SoC [2,3]. Two of the main studies that both reviews were based on were those of Behera et al. (a retrospective observational analysis (case-series) [53]) and Manci et al. [52]. According to the first study, nodular BCC dermoscopy was presented mainly with ulceration and a blue-white veil, followed by brown to blue-gray ovoid nests, while the maple leaf-like area, red-white homogenous area and multiple small erosions were presented in superficial BCC dermoscopy [53]. The correlation between dermoscopy and histopathology confirmed in Caucasian populations needs to be investigated in SoC patients [113]. According to the second study, the loss of the normal background pigmentation/network milky red area and an accentuated normal background pigmentation surrounding the lesion were also detected in the BCCs of SoC as well [52].

The dermoscopy findings of the black, gray and blue dot characteristics were found to be the most common finding of BCC according to Enechukwu et al. [3]. Black or blue-gray dots or globules or ovoid nests correspond to differently sized basal cell tumor islands in the dermis and can be frequently observed in BCCs. However, this finding can be seen in other lesions of SoC patients different to BCC, such as squamous cell carcinoma in situ (Bowen disease), apocrine hidrocystoma, nodular hidradenoma, sebaceous hyperplasia and trichilemmal cysts. This finding is less frequent in those diagnoses, while besides BCC, black or blue-gray dots in the case of Bowen disease are organized in a peripheral clustered or linear arrangement [98].

New-dermoscopy findings reported in SoC were found both in BCC and Bowen’s disease (Table 4). In BCC, the absence of a typical background pigmentation or network, a milky red region and an intensified normal background pigmentation or network encircling the lesion was a novel dermoscopy finding exclusively in SoC patients [52]. Also, in Bowen’s disease, the pattern resembled a ring, with a vague gray outer edge and a distinct, slender brown outer boundary [98]. Also, in dermatosis papulose nigra, which is a subtype of seborrheic keratosis, milia cysts were less frequently found compared to the classic seborrheic keratosis dermoscopy in Caucasians [97].

In the case of a challenging dermoscopy image, a question regarding the effectiveness of the classic algorithms in dermoscopy across diverse presentations of skin lesions arises. For example, in the case of a two-step algorithm of dermoscopy, clinicians differentiate melanocytic from nonmelanocytic pigmented lesions using a stepwise evaluation for the presence of specific dermoscopic criteria of sebaceous keratosis, dermatofibroma, BCC, squamous cell carcinoma, angiomas and sebaceous hyperplasia **[114]**. In case of SoC patients, the prevalent dermoscopy characteristics of the above-mentioned lesions are different and the diagnostic accuracy of classic dermoscopy algorithms may be disturbed. Therefore, more research is needed for the development of more universally applicable algorithms in the case of SoC patients.

## 5. Conclusions

BCCs in SoC patients can present as pigmented nodular lesions, a pigmented patch or plaque, an ulcer, an erythematous nodular lesion, an erythematous plaque or patch or a scar-like lesion depending on its subtype and its site of occurrence. The differential diagnosis of BCC in SoC patients can differ from that in Caucasians, adding to the list of skin diseases that are rare to Caucasians and removing others that appear less frequently to SoC patients. Dermoscopy is an essential diagnosis method for the detection of BCC in dark skin.

## Figures and Tables

**Table 1:** Presenting case reports, case series and studies including clinical presentation and differential diagnosis of BCC in SoC patients (type of study, ethnicity of the patient, age-gender and body site of the BCC).

Type of Study		Nationality	Location of the Lesion	Subtype	Differential Diagnosis (BCC Mimickers) Based on the Authors or Text of the Respective Study
Case report	[9]	Indian	Scalp	Nodular non-pigmented	Trauma, nevus sebaceous, radiation dermatitis
Case series	[37]	Iran	Scalp (more frequent), forehead, nose and periorbital area	Mostly nodulocystic or micronodular pigmented	SCC
Case report	[36]	NM	Vulvar	Adenoid	Extramammary Paget disease, lichen sclerosus, atrophicus and lichen simplex chronicus
Case series	[15]	NM	1o: Lower back 2o: Lumbosacral region	Adenoid	Pre-existing skin condition, indigenous drug intake (containing arsenic), exposure to irradiation or trauma
Case report	[14]	NM	Nose	Adenoid	Adenoid cystic carcinoma, metastasis
Case series	[38]	Indian	Upper lip (more frequent location) and cheek	Mostly nodular	SCC
Case report	[18]	NM	Nose	Nodular and adenoid	SCC, adenoid cystic carcinoma, scrofuloderma and deep mycosis
Case report	[19]	Indian	Scalp	Infiltrative	SCC
Case report	[20]	NM	Face	Superficial	Eczema, psoriasis, lichen planus, or Bowen’s disease.
Case series	[40]	Indian	Eyelid (mostly upper)	NM	SCC, sebaceous gland carcinoma, malignant melanoma, and miscellaneous tumors
Case series	[39]	Indian	Eyelid (mostly upper)	NM	sebaceous gland carcinoma, SCC and miscellaneous tumors
Case series	[42]	Indian	Forehead (most frequent) followed by cheeks	Mostly nodular followed by ulcerative and pigmented	SCC, melanoma, cutaneous lymphomas, and sarcomas.
Case reports	[34]	Indian	Temporal region of scalp and inner canthus of left eye	Pigmented	Melanoma, squamous cell carcinoma, discoid lupus erythematosus and nevus comedonicus
Case report	[25]	NS (Black)	Groin region	Micronodular and infiltrative	SCC, metastasis, sarcoma
Case reports	[28]	NS (Black) and Asian	Head and neck region (most frequent)	NM	SCC, dermatofibroma protuberans, nevus, melanoma
Case report	[29]	NS (Black)	Upper lip	Nodular	SCC, burn, chronic infection
Case report	[16]	African American	Nose	Morpheaform	Melanoma, seborrheic keratoses or nevus sebaceous
Case report	[31]	African American	Forehead	Infiltrative	SCC, Marjolin ulcer, metastasis
Case series	[43]	African Americans	Mostly in head and neck regions	Mostly pigmented followed by infiltrative	Seborrheic keratosis, benign nevus
Case report	[32]	African American	Eyelid	Nodular	Benign tumors, such as nevi, blue nevi, seborrheic keratosis, apocrine hidrocystomas, vascular malformations and inflammatory processes (such as a chalazion) Malignant tumors such as melanoma, metastasis, pigmented SCC
Case reports	[35]	Hispanics	1o: Nasal tip 2o: Nasal bridge 3o: Breast	Nodular	Melanoma
Case series	[49]	Chinese	Vulvar	Nodular (mostly)	Melanocytic nevus, seborrheic keratosis, malignant melanoma, SCC, adenoid cystic carcinoma

NM: not mentioned.

**Table 2:** Presenting clinical presentation and dermoscopy main characteristics of BCC in SoC patients.

BCC Clinical Image in SoC	Main Findings
Superficial	Solitary well-defined hyperpigmented plaque or patch or erythematous, indurated, irregular plaque
Nodular	Pigmented nodule (usually with pearly appearance) with or without ulcerated area or red giant nodule with or without ulcerated area
Adenoid	Mainly ulcerated lesion
Morpheaform	Scar-like lesion
Infiltrative	Mainly ulcerated lesion
BCC dermoscopy image in SoC	Main findings
Superficial	Pigmented structures such as maple leaf-like area and spoke wheel-like areas, red-white homogenous area, multiple small erosions, short fine telangiectasia, spoke wheel-like areas [2,3,52,53].
Nodular	Ulceration, blue-white veil, brown to blue-gray ovoid nests, arborising vessels [2,3,49,50,51]
Adenoid	-
Morpheaform	-
Infiltrative	-

**Table 3:** Presenting differential diagnoses according to the macroscopic image of the BCC in SoC patients.

Macroscopy Image of BCC in Skin of Color Patients	Differential Diagnosis
Pigmented nodular lesion	Cutaneous neoplasms: nevi [54], blue nevi [55], melanoma [56,57,58,59], benign appendageal tumors (such as melanotrophoblastoma and hidroadenoma, etc.) [60,61,62,63], dermatofibroma and/or pigmented dermatofibrosarcoma protuberans [64,65], vascular lesions [66] Infectious diseases: deep cutaneous mycosis (such as chromoblastomycosis in endemic regions) [67,68]
Ulcerated lesion	Cutaneous neoplasms: squamous cell carcinoma (such as Marjolin’s ulcer) [69], malignant appendageal tumors (such as hidraneosarcoma and sebaceous carcinoma) [70], dermatofibrosarcoma protuberans [71], vascular lesions [72], metastasis [73,74]
Solid pigmented plaque patch with or without ulceration	Cutaneous neoplasms: lentigious melanoma [75], pigmented Bowen [76,77], B-lymphoma [78], extramammary Paget [79], seborrheic keratosis and nevus sebaceous [80,81].
Erythematous plaque or patch	Skin cancer entities: squamous cell carcinoma and Bowen disease [82,83], lymphoma and extramammary Paget [84], Kaposi sarcoma [85] Cutaneous infections such as tuberculosis [86], chromoblastomycosis [87], cutaneous mucormycosis [88] Chronic inflammations such as lupus erythematosus [89], dermatitis [90], psoriasis and erythrokeratodermia [91,92]
Red nodule with or without ulceration	Cutaneous neoplasms: benign appendageal tumors (pilomatricoma) [93], vascular lesions and angiomas [94], melanoma [95]
Scar-like appearance	Scar, dermatofibroma [65], lichen sclerosus [96]

**Table 4:** Presenting the predominant dermoscopy findings of cutaneous neoplasms in SoC.

Cutaneous Neoplasms	Predominant Dermoscopy Finding
SCC	White areas, scales, erosions and ulcerations, polymorphic vascular pattern such as dotted or linear or irregular or serpentine vessels, white and shiny clods [52,97]
Bowen’s disease	Blue-gray dots/globules, arranged either peripherally in clusters or linearly, scales, light to dark brown keratotic structureless areas [98,99]
Dermatofibroma protuberans	Pigment network, pink background, white structureless area [100]
Dermatofibroma	Peripheral pigmented network, white patches (“central scar-like” or “eccentric multiple”), central homogeneous pigmentation [101,102]
(Acral) Melanoma	Structureless regions displaying various tones of brown, blue, black and pink colors, erosions or ulceration, parallel and fibrilla ridge pattern of surrounding skin [52]
Cutaneous lymphoma	-
Extramammary Paget	-
Sebaceous keratosis	“Moth eaten” borders, comedo-like openings, milia-like cysts, “fat fingers”, cerebriform pattern, “finger print” pattern, surface white scaling [97]
Nevus comedonicus	Not enough data to conclude to predominant dermoscopy findings. Only case reports found
Nevi (reticular)	Brown or black color, reticular lines, structureless areas [103,104,105]
Blue nevus	Homogenous structureless blue [106]
Hemangioma—vascular lesions	Red-purple clods-lagoons, white lines [97]
Skin adnexal lesions	Examples: Trichoepithelioma: white homogenous area with milia-like cyst, linear, arborizing and crown vessels Nodular hidradenoma: a white to gray structureless area, erosion and a polymorphous vascular pattern [107]
Nevus sebaceous	Papillary to knob-like arrangement on a background ranging from yellow to gray, yellow-white homogeneous region, ovoid nests [107]
Metastasis	-
Inflammation diseases	
Dermatitis (in the context of radiation)	Irregularly distributed, predominantly yellow but also including brown and white scales, purple dots and fabric fibers, brown-black dots against a background, erosions [108]
Psoriasis	Dotted vessels, diffuse or patchy distributed white scales, pigmented structures, such as brown, gray, and blue structureless areas, dots, or globules [109]
Lichen planus	Blue globules and Wickham striae [109]
Facial lichen planus pigmentosus	Brown dots/globules, pseudonetwork, loss of vellus hair [108]
Chronic lichen sclerosis	Not enough data to conclude to predominant dermoscopy findings. Only case reports found
Infectious diseases	
Deep fungal infections Examples: chromoblastomycosis	Not enough data to conclude to predominant dermoscopy findings. Only case reports found
Lupus vulgaris	Yellow-orange structureless areas, linear/dotted vessels, white scales, white structureless areas [110]

**Table 5:** Presenting the correlations between dermoscopic features in a lesion resembling BCC and its differential diagnosis on a dark-skinned patient.

Dermoscopic Features of the Lesion in SoC	Differential Diagnosis
Ulceration	BCC, SCC, melanoma, deep fungal infection (chromoblastomycosis)
Blue-white veil or blue color	BCC, melanoma, blue nevi
Ovoid nests	BCC, nevus sebaceous
Arborising vessels	BCC, adnexal tumors (melanotrichoepithelioma)
White homogenous/structureless areas	BCC, SCC, dermatofibroma protuberans, nevus sebaceous, skin adnexal tumors (nodular hidradenoma), dermatofibroma, lupus vulgaris
Scales	Psoriasis, dermatitis, SCC, Bowen’s disease, lupus vulgaris, deep fungal infection (chromoblastomycosis, etc.)
Dotted vessels	Lupus vulgaris, SCC, psoriasis
Pigment network	Nevi, melanoma, dermatofibroma protuberans
Erosions	SCC, dermatitis

## Data Availability

The data described in this study are available upon request from the corresponding author.
